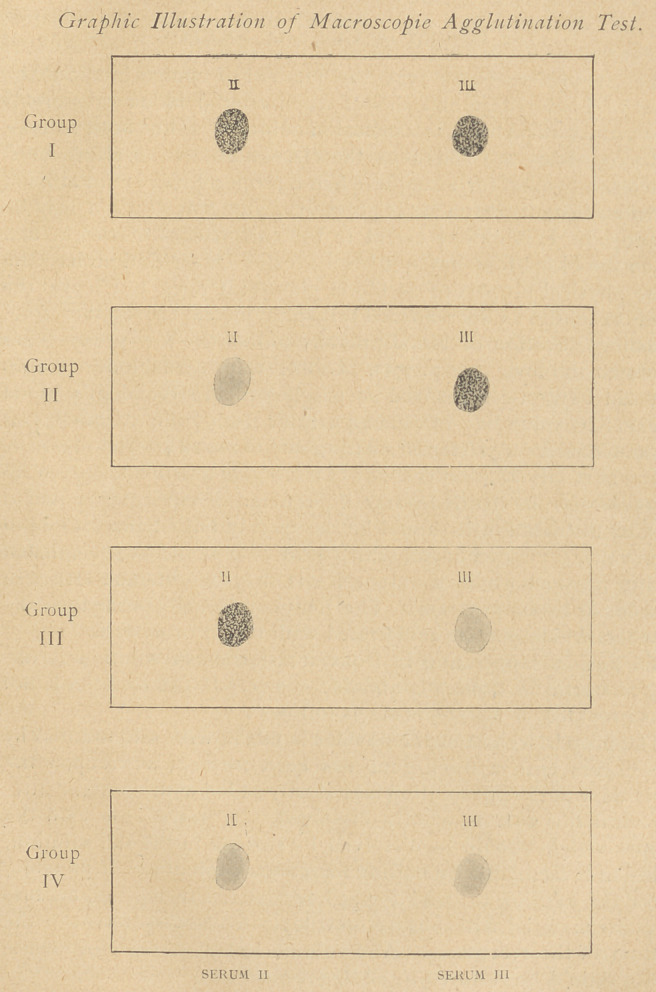# Traumatic Shock and Hemorrhage1.In this pamphlet is included the information contained in memoranda numbers 9, 10, 11, 13,15 and 17, issued from the Division of Laboratories, A. E. F.

**Published:** 1918-12

**Authors:** 


### TRAUMATIC SHOCK AND HEMORRHAGE[1]

The condition of shock which not infrequently accompanies serious wounds has been one of the most mysterious phenomena presented in surgery. It is characterized by pallor, rapid pulse, sweating, thirst, superficial rapid respiration, and a typically low blood pressure. Many theories have been offered to account for these conditions, particularly the low blood pressure — such as failure of the vasomotor center, general exhaustion of nervous centers, and pulmonary fat emboli. There is general agreement that an essential factor is stagnation of blood (exemia) somewhere in the body, so that the return of blood to the heart is insufficient to keep up the arterial pressure. Shock presents many of the clinical features resulting from severe hemorrhage : in both there is inadequate circulation of blood; in both damage to important functions results from the circulatory derangement; and in both the central problem is the restoration of an adequate blood flow.

In men suffering from shock and hemorrhage there is a concentration of the blood in the capillaries, indicated by a marked difference between the capillary and the venous blood counts. The corpuscles in capillary samples may exceed those of venous samples, taken simultaneously, by as much as 2,500,000 per cubic millimetre, a difference which indicates that the "lost blood" of shock (the exemia) is to a considerable degree accumulated in the capillary bed.

Exposure to cold has a marked effect in inducing or augmenting the state of shock. A badly wounded man may leave the front line in fair condition, but, during his journey to the rear, loss of body heat may be a decisive factor in reducing him to a state of shock. His condition is usually improved when he is placed in a warm bed and supplied with sufficient heat to restore his normal temperature. Cold, which is known to cause stagnation of blood in capillaries, increases the "lost blood" by retarding capillary flow.

### The Importance of Keeping the Wounded Warm.

The severely wounded man sweats to a degree that may even wet his clothing; he may have a diminished blood pressure from

1. In this pamphlet is included the information contained in memoranda numbers 9, 10, 11, 13, 15 and 17, issued from the Division of Laboratories, A. E. F.

hemorrhage or on-coming shock, and he is liable to additional exposure during the examination of his wounds and in transportation. All these conditions lead to lowering of body temperature and thus to lessening of the chances of survival. To preserve or to restore normal body temperature, the following precautions should be observed :

In examining wounds, expose one part at a time, carefully covering with a blanket the examined portion before proceeding further.

Badly wounded men, suffering from shock or hemorrhage, are usually thirsty. Give them hot drinks, preferably hot coffee or tea. There is good evidence that water absorbed by the digestive

Interpretation of Fig. 1.

Method of folding 3 blankets to provide four layers beneath and two above the patient.

tract may serve effectively to restore blood volume after hemorrhage.

Avoid giving drinks if there is an abdominal wound, since there is always possible penetration of the stomach or intestines.

Use three blankets if there is danger from cold. To obtain the the best service from them (4 layers) fold as shown in Fig. 1. Blanket 3 should be folded under the patient's feet. A waterproof sheet around the blanket will check loss of heat. Always use it in wet weather.

In placing hot water bottles keep in mind two considerations : that the rapidity of heat conduction depends on difference of temperature, and that more value is obtained from a hot water bottle by using both sides. For quick heating and for comfort, apply the warmth preferably to the coldest parts, i. e., to hands and feet. If only two hot water bottles are available, put one at the feet, the other on the belly with the hands over it. If three more are available, place them between the arm and chest on either side, and between the thighs.

In a " push ", when supplies may be insufficient, canteens temporarily taken from a salvage heap may be used as hot water bottles.

Be careful to avoid a degree of heat that will burn the skin.

In ambulance dressing stations, during examination of the severely wounded, the stretcher should be placed on trestles and warmed from beneath. An oil stove or can of solid alcohol set in a tin box (9 × 9 × 14 inches), which is opened *on both sides* at the top (see Fig. 2), will supply heat. Lay blankets over the trestles; allow the free portions of the two blankets under the patient to hang down from the stretcher and thus form a hot air chamber. The shocked man should not be sent further on his journey until warmed.

At the hospital undress the patient, and make him comfortable in bed. If he is cold, place over him a fracture frame, cover it

Interpretation of Fig. 2.

Method of cutting a tin can (9 × 9 × 14 inches) to make a heater. The can is supported to bring opening B opposite the space between the cot and stretcher..

with blankets, and lead into the space thus formed a stream of heated air. A stove-pipe with an elbow, if set on an oil stove and arranged so that it opens under the blankets at the foot of the bed, will make a satisfactory means of delivering heat. In place of a stove-pipe the tin box mentioned above may be cut in the manner illustrated in Fig. 2, and supported so that the upper opening connects with the space under the blankets. A can of solid alcohol set in the box, or an oil stove or an alcohol lamp beneath the box (the bottom being removed) provides a source of heat. The flame from the solid alcohol may be controlled by partly covering the can. Lay on top of the box a board to protect the blankets from being scorched. In a stationary hospital with electricity a series of electric lamps may be fastened to the fracture frame, and employed to supply heat. The frame, set over the patient and covered with blankets, is ready for use.

In case of severe shock, when the handling of the patient should be minimal, he may be left temporarily on the stretcher and warmed. The stretcher should be placed on the cot or bed and the space between them, enclosed by blankets, heated from the stove-pipe or tin box in the manner described. If the patient's clothes are wet, he should always be undressed before warmed.

*Do not overheat the patient.* He needs fluid and should not be caused to lose it by unnecessary sweating.

### The Critical Level of a Falling Blood Pressure and its Modification by Hemorrhage.

Clinical and experimental observations have shown that death after severe hemorrhage need not be immediate, but may occur after the lapse of some hours. This fact is explained by the gradual damage of essential organs by deficient circulation, due principally to reduced blood volume, until they fail to perform their functions.

If hemorrhage is severe, the oxygen supply of the tissues becomes insufficient, and an increased production of non-volatile acid (lactic) will occur. Other conditions which markedly lessen the oxygen supply to the tissues (CO poisoning, rebreathing expired air) have the same effect. Lactic acid thus produced unites with the sodium of the sodium bicarbonate in the blood, drives off the $CO^2$, and thereby produces a reduction of the " alkali reserve " (indicated by a diminished capacity of the plasma to take up $CO^2$). When the $CO^2$ capacity is reduced to less than 50 volumes per cent., under standard conditions, " acidosis " is said to be present. Reduction of the alkali reserve, in such circumstances, may be taken as an indication of insufficient oxygen supply to the tissues.

As arterial blood pressure falls, the rate of circulation of the blood decreases. Then, though the red blood corpuscles leave the lungs normally laden with oxygen, they may not bring a normal supply to the tissues because they move too slowly. In that case the condition would be similar to other conditions in which oxygen want exists; non-volatile acid would result and the alkali reserve be lessened.

In traumatic shock the blood pressure is low, and the circulation is therefore sluggish. For therapeutic purposes it is important to know at what point in a falling blood pressure the oxygen supply begins to be insufficient, as indicated by a lessening of the alkali reserve.

Experiments in the Division of Surgical Research at the Central Medical Department Laboratory have shown that if arterial pressure is lowered to 80 mm. of mercury for an hour, the alkali reserve is not reduced; but if lowered to 70 mm. the reserve begins to fall; and if lowered to 60 mm., it falls still faster (i. e., the oxygen supply is less adequate, and the production of non-volatile acid is more rapid). A critical level of oxygen supply to the tissues is reached, therefore, when the arterial pressure is experimentally lowered to less than 80 mm. of mercury.

Average figures from 43 cases of shock and hemorrhage in men studied in the summer of 1917 at a Casualty Clearing Station in Béthune reveal similar relations :

| Systolic blood pressure | Average $CO^2$ capacity | No. of cases. |
|---|---|---|
| — | — | — |
| 90–100 mm. mercury. | 49 vols. per cent. | 12 |
| 80- 90 — | 49 — | 5 |
| 70- 80 — | 43 — | 10 |
| 60- 70 — | 36 — | 11 |
| 50 - 60 — | 24 — | 5 |

As these figures clearly show, a reduction of the alkali reserve below 50 volumes per cent $CO^2$ capacity, or a condition of " acidosis ", occurred when the systolic pressure was lower than 80 mm. mercury; and the reduction of the reserve was progressive as the pressure was progressively lower. It appears, therefore, that the *critical level* of systolic arterial pressure for proper oxygen supply to the tissues is approximately 80 mm. of mercury. The lower the pressure below that level, the less is the circulation able to meet the needs of the tissues.

Experiments show, as might be expected, that if hemorrhage complicates a low blood pressure the critical level is higher than if no loss of blood has occurred. Thus, if 20 per cent. of the blood has been lost, the pressure cannot be lowered to 80 mm. without indications of insufficient oxygen supply to the tissues.

Since any muscular activity increases the demand of the tissues for extra oxygen, the patient who is suffering from the low blood pressure of shock or hemorrhage should be kept quiet. The tissue or cellular acidosis will thus be reduced to a minimum. Morphine in sufficient doses will prevent restlessness. Its value in lessening pain during transportation is too obvious for emphasis.

Nerve cells are especially sensitive to oxygen want. Continuance of blood pressure below the critical level results in damage to the control of the circulation, particularly to the nervous factors. The longer the duration of the low pressure, and the lower the pressure, the greater the damage. In time the damage may be so great that no treatment will permanently raise the blood pressure... circulatory control has been lost.

A low arterial pressure should be raised, not by damming up the already reduced volume of blood in the arteries — as results from general vasoconstriction — but by increasing the volume of circulating fluid. This is best done by transfusing blood, for thus the rate of circulation is increased by raising the head of pressure in the arteries, and oxygen carriers are also added to the circulating fluid. In the absence of blood the gum acacia solution, devised by Bayliss, should be employed. By increasing the blood volume it raises arterial pressure and accelerates the blood flow. Blood transfusion is the method of choice in treating shock and hemorrhage, and after extensive hemorrhage it is especially urgent.

From the foregoing considerations the following inferences regarding treatment are drawn :

*Morphia should be given in sufficiently large doses to keep the patient comfortable and quiet.*

*Persistence of arterial blood pressure below 90 mm. of mercury (systolic) for more than half an hour, without sign of improvement, in spite of rest, quiet, warmth and fluid, should call for transfusion of blood or infusion of gum-salt solution. If the pressure is much below 90 mm., e.g., 50 or 60 mm., such treatment should be instituted immediately. Certain cases in which the blood loss has been extreme may require a second transfusion before a satisfactory condition is reached.*

*When there is danger of internal bleeding, if the blood pressure is raised by intravenous injections, start the injection when the surgeon is ready to check any hemorrhage that may result.*

*Before operation it is best to give a shocked man time to recover both from his low blood pressure and from the effects of the low pressure.*

*In case of concealed hemorrhage, however, or rapidly spreading infection (e. g., gas gangrene), operation may be necessary before such recovery has occurred. Since ether anæsthesia is likely to lower an already low pressure, it should be given only as the pressure is being artificially raised. Blood transfusion or intravenous injection of gum-salt solution should be started, under these circumstances, as soon as anæsthesia is induced. The pressure may thus be prevented from falling during the operative procedure.*

*Whether blood for gum-salt solution has been injected or not, the patient should be given large quantities of fluid by the digestive tract for 24 to 48 hours afterwards.*

Transfusion or infusion should be repeated after the operation if blood loss has been considerable, or if any other indication, such as persistent low blood pressure, appears.

It should be remembered that cases in which low blood pressure has persisted for a long time may improve only slowly after treatment.

Positive indications for transfusion in secondary anemia, with prolonged suppuration and sluggish wound healing, will be found in repeated blood counts and hemoglobin determinations. Repeated transfusions are often necessary before the desired improvement is attained.

Since the transfusion of blood can be of so much benefit to the recipient, and is so slight a tax upon the donor, surgical officers and officers in charge of resuscitation of surgical patients should realize that, whereas an error of omission may cost a life, an error of commission can cause but slight transient discomfort.

If through unavoidable circumstances a low blood pressure has persisted for so long a time as to induce an extreme acidosis, indicated by rapid, deep respiration ("air hunger"), intravenous injection of 3 per cent. sodium bicarbonate may give marked and immediate relief. Such treatment should be followed by other methods to raise arterial pressure.

In cases of secondary hemorrhage, treatment should be determined in accordance with the urgency of the patient's condition. With pallor, and small rapid pulse, and blood pressure below the critical level, transfusion should be given. Less urgent cases may be satisfactorily treated by giving as soon and as rapidly as possible large quantities of fluid by mouth, and salt solution by rectum.

### The Method of Transfusing Blood.

In March, 1918, a committee representing the laboratory and surgical services of the United States Army adopted transfusion with citrated blood as the method for combatting shock and hemorrhage in the hospitals of the American Expeditionary Forces[1]. The reasons for this choice were simplicity of equipment and technic, convenience to donor and recipient, and excellence of results. The chief precaution to be regarded is the quick delivery of the blood through clean tubes into the citrate solution, so that changes in the blood in the direction of coagulation may be arrested as soon as possible.

The only apparatus required for this method is a litre bottle

(1) See "A Report upon the Transfusion of Blood for the Recently Injured in the United States Army", published by the Medical Division of the American Red Cross Society in France, 4, place de la Concorde, Paris, May, 1918. The present description is taken largely from that Report.

provided with two rubber stoppers having two perforations, appropriate glass and rubber tubing, and two transfusion needles. (See Figs. 3 and 4.) The largest size needle is used for bleeding, the small size for giving the blood. The rubber tube B should be short and of large bore to assure a rapid flow and lessen the chance of coagulation. A convenient suction and pressure pump may be made from an ordinary Davidson syringe. Suction or pressure can be made by reversing the ends.

The bottle E and the stoppers and tubing are wrapped in a towel and sterilized in an autoclave. Prepared in this way, the apparatus may be kept sterile and ready for immediate use. If an autoclave is not available, the apparatus should be sterilized by boiling in previously boiled or in distilled water.

The needles are sterilized by boiling just before the transfusion. If they are being repeatedly used, they may be sterilized in boiling liquid petrolatum or albolene, and left standing in the oil until needed.

Interpretation of Fig. 3.

A. Transfusion needle. — B. Rubber tube. — C. Glass tube. — D. Rubber stopper. — E. Bottle of litre capacity. — F. Glass tube. — G. Rubber tube. — H. Glass tube for suction (cotton in bulb).

The needle is the most important part of the apparatus and requires careful attention. Before each bleeding it should be well sharpened. The chief consideration in the sharpening is to produce a fine spear point with a bayonet edge. This is best done by grinding first on the bevel — which should be moderately short — and then on the back of each edge at the point. If the point is well protected when not in use, sharpening will require only a few moments. Before boiling, the needle should be slipped into a short length of rubber tubing. The needle must be kept scrupulously clean. After each bleeding it should be washed out at once, all fragments of fibrin or clot removed from the base, and small pieces of cotton soaked in oil thrust through the lumen with the stilette. The whole needle should be well oiled before being put away

Before the blood is collected, a tube of sodium citrate is broken off at the file mark, the opened end flamed, and the contents poured into the bottle E. Normal saline solution (0.9 o/o) is then added up to 100 cc., (i. e., to the *top* of the figure). When the bottle is filled to 700 c c. the citrate present is 0.21 per cent. The apparatus (see Fig. 3) is then assembled so that the rubber stopper fits snugly into the mouth of the bottle. Great care should be taken to keep all the open parts sterile.

*Bleeding* : The donor's arm is now extended at a right angle to the body. A tourniquet is applied to the arm high up — the cuff of a blood pressure apparatus folded to half its width makes an excellent tourniquet with the pressure kept at 50-60 mm. of Hg. Choose a suitable vein in the bend of the elbow, remembering that the needle is best inserted toward the hand. It is important to have as large a vein as possible. Opening and closing the fist and flicking the skin over the veins cause them to dilate considerably. The tourniquet is then released. The skin over the vein is scrubbed with soap and water, and the sterilization completed with alcohol. At the point selected for venepuncture a small quantity of novocaine or cocaine is injected intracutaneously. A very slight nick is then made throught the skin with the point of a scalpel. The tourniquet is tightened and the means above described are again employed to dilate the vein. Do not touch the point of puncture. The bottle is placed on a stand close to the patient's arm in such a position that there will be no kinking of the tube B when the needle is in the vein. After drying the skin-opening, with a piece of sterile gauze, the needle is inserted for a short distance beneath the skin, then by raising the base slightly it is pushed into the vein. It is essential to keep the needle immobile. The operator should hold it throughout the bleeding, steadying his hand against the donor's arm. With the free hand the bottle is given a rotary motion every few seconds in order to insure thorough mixing of the blood with the citrate, which is very important. A moderate degree of suction is maintained either by means of the tube H, which is held in the operator's mouth, or more conveniently by using the adapted syringe-pump. The donor continues to open and close his hand slowly, making a firm fist each time, care being taken that he does not move his arm.

The citrated blood does not coagulate and consequently its introduction into the recipient need not be hurried. Under ordinary conditions the blood will be used immediately, but when occasion requires it may be kept for several hours before introduction. If, in the course of drawing the blood, clotting occurs and the blood ceases to flow, release the tourniquet, withdraw the needle and obtain the blood through use of entirely fresh apparatus (needle, rubber, and glass tubing) which should be at hand sterilized for such an emergency. It is usually better to take the other arm.

Six hundred (600) cc. of blood is the limit to be removed, for a donor may lose this amount without distress. If more blood is required a second donor must be taken. The same donor must not be used twice within a single week.

The bottle of blood should be placed in a receptacle containing water at about body temperature, where it should be kept during the transfusion.

*Transfusion :* The introduction of the blood into the recipient is accomplished by removing the first stopper D (Fig. 3) with its connections and putting the stopper N (Fig. 4) with its connections snugly into the mouth of the bottle. Air pressure is increased by blowing through the tube Q, and blood begins to rise in the tube M, which forms one limb of the siphon K, L, M (Fig. 4). The tube K is held high as the blood passes into the rubber tube L, and then is gradually lowered. When K is completely filled, a pinch-cock closes the rubber tube L close to the glass tube K.

Interpretation of Fig. 4.

I. Transfusion needle. — J. Rubber tube. — K. Glass tube. — L. Rubber tube. — M. Rubber stopper. — O. Glass tube. — P. Rubber tube. — Q. Glass tube for exerting compression (cotton in bulb).

A bandage or tourniquet is placed about the arm of the recipient sufficiently tight to give the maximum venous pressure. Remember that the arterial pressure of the recipient is low; the arterial flow must continue if the veins are to be made prominent. The needle I with the short rubber tube J attached is then introduced, in the direction of the venous stream, into the vein of the recipient. As the blood begins to flow through the needle and tube, the assistant quickly removes the bandage while the operator immediately connects the rubber tube J with the glass tube K, the precaution being observed to have *both tubes filled with blood.* The bottle is then raised to the full height allowed by the rubber tube L, the pinch-cock is opened, and the blood enters the recipient by gravity. The time allowed for the introduction of 600 cc. of blood should not be less than 10 to 15 minutes. Any symptoms of distress should indicate a checking of the flow. Such symptoms, which are usually nothing more than a feeling of fulness and slight respiration difficulty, are ordinarily transient. At the completion of the transfusion a small amount of blood will remain in the bottle below the level of the glass tube M.

If more convenient, the bulb of a Davidson syringe or of a blood pressure apparatus may be connected with tube P (Fig. 4), and the blood forced in by air pressure.

If the veins of the recipient are very small or collapsed, an incision may be made and a canula introduced into the vein.

After use the apparatus must be cleansed with cold water immediately. If not being frequently used, the needles should thereupon be dried by running first alcohol and then ether through them, after which they should be stored in test-tubes with a cotton plug in the bottom and the mouth of the tube. The needles must be kept sharpened.

### The Selection of Donors.

There exist in the plasma of animals certain bodies which will agglutinate or agglutinate and hemolyse the red blood cells of other individuals who are members of the same species. The transfusion of such incompatible blood may be fatal to the recipient. Among human beings it is definitely known that all individuals fall into one of four groups. Knowledge of these groups has proved of practical value in blood transfusion. Hemolysis does not take place between individuals belonging to the same blood group, and practically never takes place between certain definite combinations of different groups. Having determined the blood group, it is possible to select a donor whose blood is compatible, as regards hemolysis, with the blood of the recipient.

The classification of these groups is as follows :

Group I. Serum agglutinates no corpuscles. Corpuscles agglutinated by sera of Groups II, III and IV.

Group II. Serum agglutinates corpuscles of Groups I and III. Corpuscles agglutinated by sera of Groups II and IV.

Group III. Serum agglutinates corpuscles of Group I and II. Corpuscles agglutinated by sera of Groups III and IV.

Group IV. Serum agglutinates corpuscles of Groups I, II and III. Corpuscles are not agglutinated by any serum.

The incidence of the four groups is approximately :

Group I, 5 o/o; — Group II, 40 o/o; — Group III, 10 o/o; — Group IV, 45 o/o.

The following table shows the relation of the four blood groups with respect to agglutination of corpuscles :

| | Serum | | | |
|---|---|---|---|---|
| Corpuscles | I | II | III | IV |
| I . . . . . . . . . . . . | o | + | + | + |
| II . . . . . . . . . . . | o | o | + | + |
| III. . . . . . . . . . . | o | + | o | + |
| IV. . . . . . . . . . . | o | o | o | o |

(+ = agglutination, o = no agglutination).

In order to determine the group of an individual, it is sufficient to test his corpuscles against known sera of Groups II and III. This is readily accomplished by a macroscopic test, which in addition to the two known sera requires only a glass slide, a needle, and two small glass rods. Citrated sera for this test are furnished by the Central Medical Department Laboratory. These sera remain active indefinitely, as a rule, but they should be tested occasionally against blood of known groups to prove that they are active and ready for emergency.

The test is performed as follows :

By means of the stopper in the bottle place a drop of Group II serum on the left half of the glass slide (slide need not be sterile, but should be clean and dry) and a drop of Group III serum on the right half of the slide.

Puncture the ear or finger of the individual to be tested, and transfer in turn to each of the sera about 1/3 of a drop of blood, on the end of the glass rod, mixing the blood intimately with the serum. Avoid mixing too much blood with the serum; it will prevent a clear result. Take care to transfer the blood before coagulation has commenced. Avoid mixing the two sera; a separate glass rod or opposite end of a rod must be used for each transfer. Agitation of the slide accelerates the appearance of an agglutination.

Within a few seconds after mixing the blood and sera one may see a brick-dust-like appearance in one or both sera, or one may see only a homogeneous suspension of the cells in one or both sera. If the distinction between the " brick-dust " and the homogeneous appearance should not be quite clear, tip the slide towards the vertical; a thin layer of blood will be left in the upper limits of the drop in which the difference, if present, will be evident. The brick-dust-like appearance denotes agglutination. Occasionally there is a tendency to rouleaux formation, which may be confusing. Rouleaux formation appears more slowly than agglutination and, contrary to agglutination, is dissipated if the rouleaux are broken up by stirring the serum. In the rare instances in which the agglutination is questionable, the donor should not be used.

Groups are indicated as follows:

When agglutination occurs in both sera the individual belongs to Group I.

When agglutination occurs *only* in III serum the individual belongs to Group II.

When agglutination occurs *only* in II serum the individual belongs to Group III.

When *no* agglutination occurs in *either* serum the individual belongs to Group IV.

Except in cases where the risk of delay is greater than the risk of hemolysis, the compatibility of the blood of donor and recipient should be determined before transfusion. It is not necessary that the donor belong to the same group as the recipient. The only practical consideration is that the recipient does not agglutinate the red corpuscles of the donor. From the above table it is seen that the red cells of Group IV are not agglutinated by the serum of any other Group. Therefore, in practice it is simpler, whenever possible, to use only donors of Group IV, in which case the patient's blood does not require testing. Group I recipients can take donors of any group, since the serum of Group I agglutinates the cells of no other group. Recipients of the other groups can take donors of their own group or Group IV only.

No person should be used for a donor who has, or has had, syphilis, malaria, trench-fever, or who has recently recovered from other infectious diseases.

Lightly gassed patients, i. e., patients whose color is normal or nearly normal, may be used as donors if properly grouped and free from transmittable disease.

Patients with scabies may be used as a source of blood for transfusion if they are otherwise satisfactory.

In general, convalescent patients who are non-febrile and in good physical condition constitute the class from which donors may be selected.

No reward is to be offered a donor; his consent must be obtained without urging or compulsion.

A list of donors, with their group, age, ward and bed, must be posted in the operation room and in the resuscitation ward.

When necessity arises, a donor is thus immediately obtainable. To avoid a possibility of error this list should provide every means

*Graphic Illustration of Macroscopie Agglutination Test.*

for proper identification. For absolute assurance, small perforated metal tags are provided, marked to indicate the group to which the man belongs. This tag must be attached to the man by the individual making the test at the time the grouping is determined.

### The Infusion of Gum-Salt Solution.

As stated above (see p. 775) the method of choice in the treatment of hemorrhage and shock, when arterial blood pressure is below the critical level, is transfusion of blood. Thus both volume and corpuscles are added to the circulation. In certain emergencies, however, for example when a proper donor is not at hand or when instant treatment is necessary, gum-salt solution may be employed. It is chiefly useful in the early stages of shock, and when the blood loss has not been great. This solution has the same physical properties as blood plasma; it does not leave the vessels rapidly, as does normal salt solution, and causes, therefore, a lasting elevation of blood pressure unless the low pressure has persisted too long (see p. 775). Unlike transfused blood, it does not add oxygen-carriers (red corpuscles) to the recipient's blood stream; its value lies in causing greater use of the corpuscles as oxygen-carriers by faster circulation due to raised arterial pressure.

The gum-salt solution consists of 6 o/o *gum acacia* and 0.9 o/o *sodium chloride*, in distilled water if possible. If tap water is used in making the solution it should be thoroughly boiled and filtered to remove calcium carbonate, which otherwise would be precipitated in the finished product. Gum acacia may be obtained either in powder or in lumps (tears). The lumps are usually purer than the powder, which may be adulterated with starch or dextrin.

To hospitals operating in front areas gum-salt solution is furnished sterile in bottles containing 500 cc. If the solution is not thus provided, it may be made as follows:

The proper weight of the powder is mixed with salt solution by the fingers until a thick gum has been formed, whereupon the remainder of the salt solution is gradually added with constant stirring until the gum is wholly dissolved. If "tears" are used the proper weight should be dissolved by stirring in the salt solution. Solution is facilitated by boiling for about 5 minutes. If possible, allow the solution to stand for 24 hours after the boiling, because resinous material will thus become deposited as a sediment in the bottom of the receptacle. Any volume lost as a result of evaporation should be restored by adding water.

To filter the solution, pass it through absorbent cotton and then through coarse filter paper. The layer of absorbent cotton may be split in two and these laid criss-cross in order to make a closegrained filter. The cotton should reach to the rim of the funnel and should be supported by a layer of gauze. The cotton is prevented from floating by small glass stoppers laid in the cone of the filter. Let the solution run *twice* through the same filter. If suction can be used, it will facilitate the filtration. The passage of the solution through double filter paper of coarse texture is the final step in its clarification.

Solutions of gum acacia in normal saline have an acid reaction. They should be neutralized before use. To titrate, take 10 cc. of the solution, dilute approximately to 25 cc. with distilled water and titrate with 1/10 normal NaOH, using phenolphthalein as an indicator. Now neutralize the whole solution by adding the proper calculated amount of normal NaOH. *The solution must not be alkaline.*

To sterilize the solution, place it in flasks or in bottles of hard glass, closed by a layer of cotton completely surrounded by gauze, and autoclave it at 120° for at least a half hour. A layer of cotton covered with gauze, previously tied about the mouth of the receptacle, will provide at any time a clean lip over which the solution may be poured. Allow the bottles to cool in the autoclave.

The sterile solution is warmed to about 110° Fah. in order to reach the vein at about body temperature, and is injected by the methods commonly used in saline infusions. The bottle and tubing used for transfusing blood (see Fig. 4) may be employed. It has been found that 500 cc. is usually an adequate amount to cause a satisfactory rise of pressure (arterial), but this amount may later be repeated if circumstances require it. Use of gum-salt solution does not preclude a later transfusion of blood if that seems desirable.

*Inject the gum-salt solution slowly.* About 20 minutes should be required to introduce 500 cc.

### Surgery in Relation to Shock.

Operation on a man who is in shock, or who has been for a considerable period in shock and has to some degree recovered, is likely to be hazardous, because the already low blood pressure may be seriously reduced in consequence of operative procedures. A number of conditions contribute to this danger, some of which can be avoided.

*Anesthesia*: Clinical observations have shown that after the body has been damaged by a shock blood pressure, there is great sensitiveness to ether and chloroform anesthesia. Experimental tests have proved that a degree of anesthesia which abolishes in a shocked animal the simple reflexes may cause the arterial pressures to fall rapidly 20 mm. of mercury or more. In a series of human cases the fall of pressure during operation averaged 30 mm. of mercury — a disastrous drop in view of the already existing low pressure. There are two ways of avoiding this harmful change ; by use of nitrous oxide and ogygen as an anesthetic, and by sustaining the pressure if ether is employed.

Clinical and experimental observations have demonstrated that if anesthesia with nitrous oxide and oxygen is properly produced a shock blood pressure need not be lowered at all during the course of operation. Pre-operative administration of morphine should be followed by *expert* use of nitrous oxide and oxygen in the ratio of not more than 3 parts nitrous oxide to 1 part oxygen. A higher ratio may cause as great a fall of pressure as is produced by ether. Deep anesthesia and cyanosis are to be avoided at all times. The surgeon must adjust himself to this light anesthesia, and its consequent absence of complete relaxation, by patience and gentleness and by a larger operative incision when necessary.

If nitrous oxide and oxygen are not available, ether given by the drop or vapor method should be employed. As soon as the anesthetic is started, however, a blood transfusion or an infusion of gum-salt solution should be started and allowed to continue slowly. The head of arterial pressure is thus maintained and may even be raised during the period when it otherwise would be much lowered.

Chloroform and ethyl chloride, which are even more depressant to the circulation than ether, are to be employed only when no other means of producing anesthesia is obtainable.

The foregoing directions are approved by the Chief Consultant in Surgery of the American Expeditionary Forces.

*Heat* : The same effort to prevent heat loss should be made during operation on a shocked man as is urged for all earlier stages. Scrupulously avoid unnecessary exposure of the body. Do not permit skin and protective coverings to remain wet. Wash out cavities and wounds only with warm solutions.

*Manipulations* : Do not expose or pull on abdominal or thoracic viscera more than is absolutely required by circumstances. Do not force liquids into cavities under pressure. Handle all tissues with extreme gentleness.

After a laparotomy has been performed on a man who is or has been in shock, a turn of the body laterally causes a sharp drop in blood pressure. Attend to wounds of the back before opening the belly. Apply binders or many-tailed bandages by lifting the body, not by turning it to one side and the other.

The shocked man should always be handled with the greatest gentleness and care.

*Tourniquet* : There is evidence that long continued exclusion of the circulation from tissues, whether injured or normal, by a tourniquet or by constrictive dressings, will in some manner induce shock when the blood flow is restored. Such evidence renders the following procedures imperative :

Whenever an extremity is so badly shattered that amputation is inevitable, a tourniquet should be applied as distally as possible and should not be removed before amputation proximal thereto.

If possible, avoid altogether the use of a tourniquet to control hemorrhage; if one is absolutely required, it should be placed as distally as the conditions permit. This precaution is necessary because later it may be decided to sacrifice the part because of permanent injury, or because of danger to life if the tourniquet is removed, or because of the development of gas gangrene.

Every wounded man to whom a tourniquet is applied should be marked by a tag stating the time of its application and the reason.

*Co-ordination* : The resuscitation team should co-operate closely with the surgical service. Resuscitation officers who have followed the progress of shock cases from the time of admission, and who best know the limits of improvement in each instance, should give the surgeon their judgment of the optimum time for surgical intervention.

Even apparently hopeless cases should be given the chance which surgery offers, though the percentage recovery of such cases may be small.

### The Duties of Resuscitation Officers.

As far as possible a trained man is to be assigned to the transfusion work of each evacuation hospital and advanced hospital for the seriously wounded (preferably a member of the fixed staff of the hospital). The duties of such a man are :

(1). To provide continually an adequate number of donors who have been properly grouped.

(2). To group all donors and recipients.

(3). To be available for consultation with any of the hospital staff concerning transfusion.

(4). To perform or direct personally all transfusions.

(5). To supervise all transfusion records in order to add information on the subject to clinical and laboratory data.

(6). To instruct other men assigned to transfusion work.

(7). To do such other clinical work as the surgeon in charge may direct.

### Transfusion Equipment for a Hospital.

### A. — *Apparatus.*

| | | |
|---|---|---|
| 1. | Plain glass bottles of 1 litre capacity, marked 100 cc., 400 cc. and 700 cc. at the proper levels . . . . . . . . . . . | 4 |
| 2. | Rubber stoppers to fit the bottle (2 perforations) . . . . . | 6 |
| 3. | Glass tubing. The glass tubing has a total diameter of 5 mm., thickness of wall 1 mm.; opening 3 mm. (for identification see interpretation of Figs. I and II). | |
| C. | Long right angle tubes, one arm 10 cm. long, the other 6 cm. long. . . . . . . . . . . . . . . . . . . . . | 3 |
| F. O. | Short right angle tubes, each arm 6 cm. long. . . . . . . | 6 |
| H. Q. | Tube for suction or compression (dilated in center for reception of small bit of cotton), 1 cm. long . . . . . . | 4 |
| K. | Straight tubes, narrow at the top, 10 cm. long. . . . . . | 2 |
| M. | U tube, short arm 4 cm. in length, long arm 25 cm. in length . . . . . . . . . . . . . . . . . . . . . . | 3 |
| 4. | Rubber tubing. The rubber tubing has a total diameter of 6 1/2 mm., thickness of wall 1 1/2 mm., opening 3 mm. | |
| B. | Short tubing for collection needle, 12 cm. long. . . . . . | 3 |
| G. P. | Tubing for suction or compression 30 cm. long . . . . . | 3 |
| J. | Short tubing for introduction needle, 7 cm. long. . . . . | 3 |
| L. | Long tubing for introduction apparatus, 1 metre 30 cm. long | 2 |
| 5. | Transfusion needles. These are made of steel. The total length of each needle is 4 cm.; the length of the shaft is 2 1/2 cm.; the length of the point is 4 mm., the length of the base is 1 1/2 cm. with a flat portion on two opposite sides. The size of the needles and the number required are : | |

25/10 mm., 2; — 20/10 mm., 2; — 16/10 mm., 2; — 13/10 mm., 2.

| | | |
|---|---|---|
| 6. | Stone for sharpening needles. . . . . . . . . . . . . | 1 |
| 7. | Small infusion cannulae. The total length of the cannulae is 39 mm.; the length of the tip is 2 mm. and there is a small collar at the base of the tip, giving a diameter 1 mm. larger than that of the shaft; the length of the tip and shaft together is 33 mm.; the length of the base is 6 mm.; the sizes of the cannulae are : one of 20/10 mm., the other 30/10 mm. . . . . . . . . . . . . . . . | 2 |
| 8. | Pinch-cock. . . . . . . . . . . . . . . . . . . . . | 1 |
| 9. | Test tubes for needles. . . . . . . . . . . . . . . | 12 |
| 10. | Small test tubes for cannulae. . . . . . . . . . . . . | 3 |

11. Paraffin 54° C. . . . . . . . . . . . . . . . . . . . . . . . 1 kilo
12. Emery paper small sheets . . . . . . . . . . . . . . . . . 10
13. Wooden box, well made and finished, to carry above apparatus, labeled : " Transfusion, Medical Dept. ", U. S. Army.

B. Laboratory Transfusion Supplies :

1. Bottles of Group II and Group III sera, labeled as below :

| Serum II | | Serum III | |
|---|---|---|---|
| Sodium citrate. . . . . | 1.5 | Sodium citrate . . . . . | 1.5 |
| Tricresol . . . . . . . | 0.25 | Tricresol. . . . . . . . | 0.25 |
| Date . . . . . . . . . | » | Date. . . . . . . . . . | » |

1 bottle of each serum.

2. Sterile sealed tubes of sodium citrate solution with file mark for breaking point, labeled : " Sodium citrate in 0.9 saline, quantity sufficient to citrate 600 cc. of blood to 0.21 : date... " . . . . . . . . . . . . . . . . . . . . . . . . 48 tubes
3. Glass slides with II and III marked into the glass, in the upper left and right hand corner respectively. . . . . . 12
4. Small tube containing small glass rods . . . . . . . . . »
5. Small bottle of alcohol for care of needles . . . . . . . . »
6. Small bottle of ether for care of needles . . . . . . . . . »
7. Bottle of blood counting fluid. . . . . . . . . . . . . . . »
8. Small metal tags (perforated) stamped to indicate the group of the patient :
   - Blood Group I. . . . . . . . . . . . . . . . . . . 10
   - Blood Group II . . . . . . . . . . . . . . . . . . 100
   - Blood Group III. . . . . . . . . . . . . . . . . . 20
   - Blood Group IV. . . . . . . . . . . . . . . . . . 100
9. Small ball of twine for fastening tag to the individual groups.
10. Wooden box, well made and finished, to carry the above supplies, labeled :

" Laboratory Supplies
Transfusion, Medical Department ", U. S. Army.

Needles and standard bottles are difficult to obtain. Please take scrupulous care of the apparatus issued. Return to the Central Medical Department Laboratory or to advanced medical depots empty bottles for gum-salt solution. Extra parts of the transfusion sets may be obtained by requisition to the Central Medical Department Laboratory.

## Figures and Tables

**Interpretation of Fig. 1. f1:**
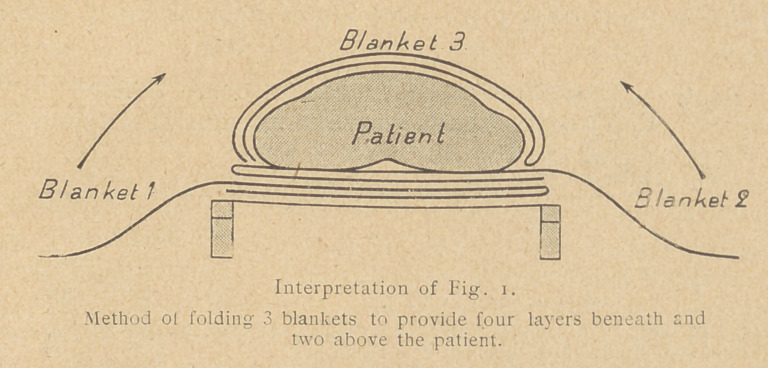


**Interpretation of Fig. 2. f2:**
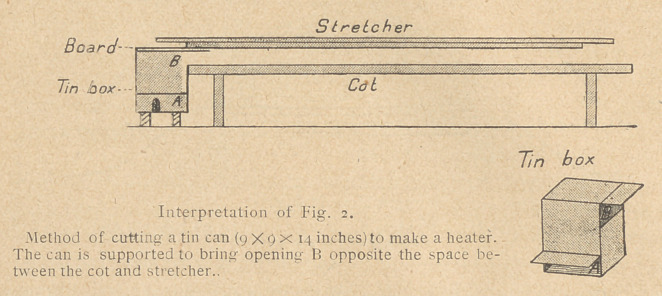


**Interpretation of Fig. 3. f3:**
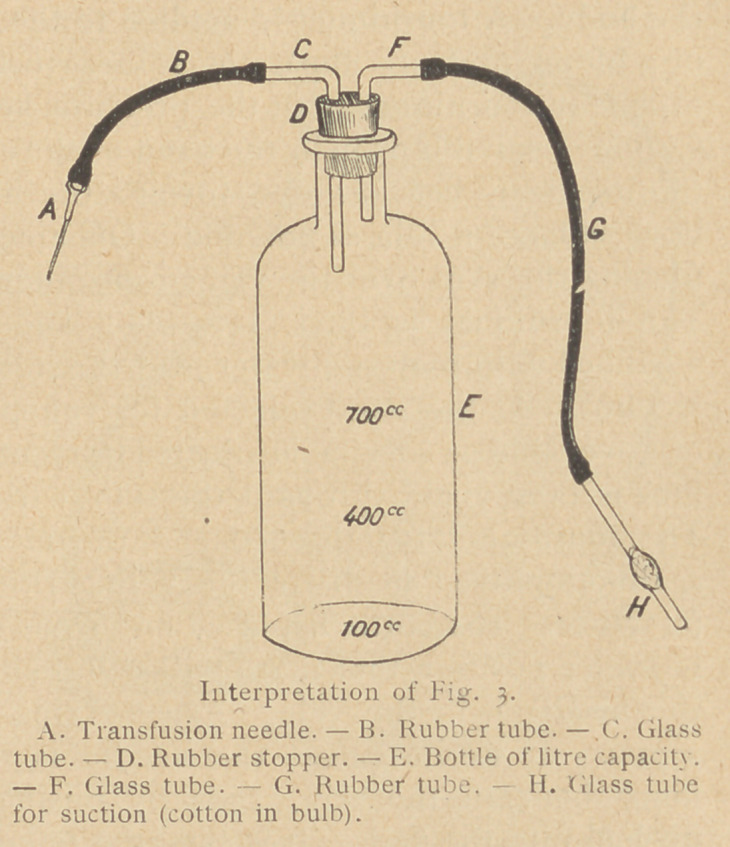


**Interpretation of Fig. 4. f4:**
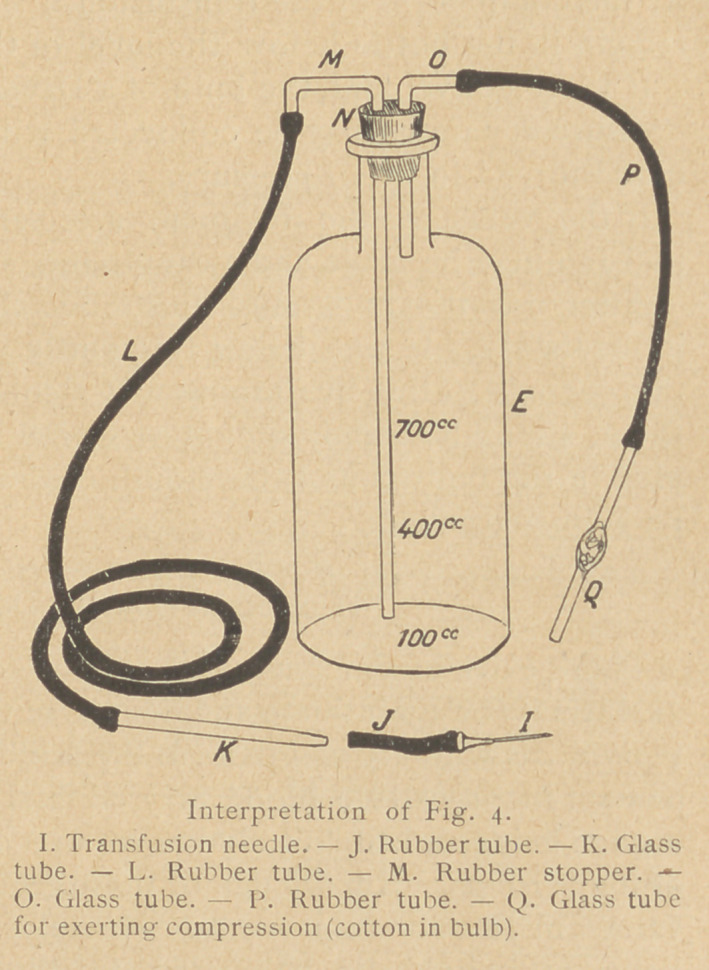


**Figure f5:**